# The Prognostic Ability of ECG Findings in Predicting Cardiovascular Events: A Five‐Year Nested Case—Cohort Study in an Iranian Population (Shiraz Heart Study)

**DOI:** 10.1002/hsr2.71830

**Published:** 2026-02-28

**Authors:** Seyed Alireza Mirhosseini, Pouria Azami, Raziye Saeedizade, Mehrab Sayadi, Mahya Beykihosseinabadi, Mohammad Keshavarz, Masood Dindari Parizi, Mahsa Borjzadehgashtaseb, Mohammadjavad Nobakhti, Armin Attar, MohammadJavad Zibaeenezhad

**Affiliations:** ^1^ Cardiovascular Research Center, School of Medicine Shiraz University of Medical Sciences Shiraz Iran; ^2^ MD‐MPH Department, School of Medicine Shiraz University of Medical Sciences Shiraz Iran; ^3^ School of Medicine Shiraz University of Medical Sciences Shiraz Iran; ^4^ Department of Cardiovascular Medicine, School of Medicine Shiraz University of Medical Sciences Shiraz Iran; ^5^ School of Medicine Qazvin University of Medical Sciences Qazvin Iran

**Keywords:** atherosclerotic cardiovascular disease, cardiovascular events, electrocardiography risk score

## Abstract

**Background and Aims:**

Electrocardiographic (ECG) markers may provide incremental prognostic value for cardiovascular disease (CVD) events beyond traditional ASCVD risk scores. This study aimed to evaluate the association between baseline ECG parameters and the incidence of cardiovascular events over 5 years in a nested case‐control cohort.

**Methods:**

We analyzed 442 participants from the Shiraz Heart Study, including 221 individuals who experienced CVD events and 221 ASCVD risk‐matched event‐free controls. Detailed ECG parameters and clinical variables were evaluated. Multivariable logistic regression, including a prespecified 22‐variable model and LASSO penalized regression, was used to identify independent ECG predictors. Model performance was assessed using bootstrap‐corrected area under the curve (AUC).

**Results:**

In multivariable analyses, ST‐segment coving (OR 7.57, 95% CI 2.09–27.4, *p* = 0.002) and shorter PR interval (OR 0.98 per ms decrease, 95% CI 0.95–1.00, *p* = 0.036) remained independent predictors of incident CVD events in the prespecified 22‐variable model, which was constructed based on clinical relevance and supported by univariable analyses. This model demonstrated excellent discrimination (bootstrap‐corrected AUC 0.97, 95% CI 0.96–0.98). In the parsimonious four‐variable LASSO model, ST‐segment coving (OR 4.31, 95% CI 1.38–13.0) and PR interval (OR 0.977, 95% CI 0.961–0.992) remained independent predictors, and model performance remained robust (bootstrap‐corrected AUC 0.96, 95% CI 0.94–0.98). Other ECG features, including prolonged QTc, abnormal R‐wave progression, left ventricular hypertrophy, and T‐wave inversions, were significant in univariable analyses but did not remain independent predictors in multivariable models.

**Conclusion:**

Baseline ECG parameters provide independent prognostic information for cardiovascular events beyond traditional ASCVD risk factors. These findings highlight the potential of ECG markers to enhance risk stratification, although external validation in larger and diverse populations is warranted before clinical implementation.

**Abbreviations**
ACC: American College of Cardiology
AHA: American Heart Association
AI: artificial intelligence
AIC: Akaike Information Criterion
ASCVD: atherosclerotic cardiovascular disease
AUC: area under the curve
AV: atrioventricular
BMI: body mass index
BP: blood pressure
CI: confidence interval
COPD: chronic obstructive pulmonary disease
CVD: cardiovascular disease
DBP: diastolic blood pressure
DM: diabetes mellitus
ECG: electrocardiogram
EPV: events per variable
FDR: false discovery rate
HDL: high‐density lipoprotein
HTN: hypertension
LASSO: least absolute shrinkage and selection operator
LBBB: left bundle branch block
LDL: low‐density lipoprotein
LVH: left ventricular hypertrophy
MI: myocardial infarction
ms: millisecond
OR: odds ratio
PAC: premature atrial contraction
QRS: QRS complex (combined Q, R, and S waves)
QTc: corrected QT interval
RAD: right axis deviation
RBBB: right bundle branch block
ROC: receiver operating characteristic
SBP: systolic blood pressure
SHS: Shiraz Heart Study
TG: triglycerides

## 1 Introduction

Cardiovascular disease (CVD) continues to be a leading cause of death and disability worldwide, imposing a significant burden on global healthcare systems [1, 2]. Despite the availability of various risk prediction models that rely on clinical parameters such as age, blood pressure, cholesterol levels, and smoking status, predicting cardiovascular events remains a challenge [3, 4]. Traditional models often overlook the valuable diagnostic potential of baseline electrocardiogram (ECG) findings, which could enhance early detection and risk stratification [5, 6]. ECG is a widely available, non‐invasive tool that can provide critical insights into a patient's cardiovascular health [7, 8]. Certain ECG parameters, such as Q waves, ST‐segment changes, T‐wave abnormalities, and QT prolongation, have been shown to correlate with an increased risk of cardiovascular events, including myocardial infarction, arrhythmias, and heart failure [9].

However, despite these associations, there remains a gap in the literature regarding the predictive value of these ECG markers over extended periods [10, 11]. Current cardiovascular risk models typically do not incorporate ECG findings systematically, limiting their ability to identify individuals at high risk for adverse cardiovascular outcomes [12]. This gap is especially critical for older populations, who are at heightened risk for both fatal and non‐fatal cardiovascular events [13]. The primary aim of this study is to evaluate whether baseline ECG variables, including QRS complex, ST‐T segment, and QT interval, can predict cardiovascular events in the Shiraz Heart Study (SHS) cohort over 5 years, alongside other established risk factors.

## 2 Methods

### 2.1 Study Design and Population

**Figure 1:**
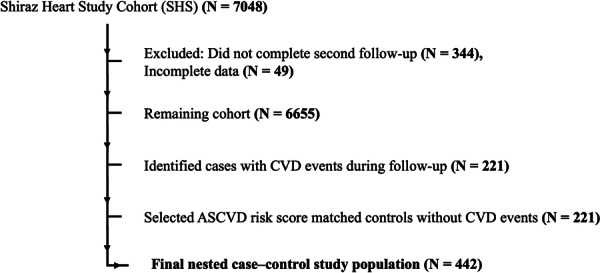
Study population flowchart. From the Shiraz Heart Study (SHS) cohort (*N* = 7048), participants who did not complete the second follow‐up (*N* = 344) or had incomplete data (*N* = 49) were excluded, leaving 6655 individuals. Among these, 221 cases with incident CVD events during follow‐up were identified. An equal number of controls (*N* = 221) were selected, matched by ASCVD risk score. The final nested case–control study population consisted of 442 participants.

This study is a nested case‐control study within the Shiraz Heart Study (SHS) prospective cohort, a community‐based study focused on cardiovascular health among residents of Shiraz. Cases were participants who experienced CVD events during the 5‐year follow‐up, and controls were selected from the remaining cohort participants who remained event‐free, using 1:1 propensity score matching on baseline ASCVD risk score. Inclusion criteria were participants in the SHS cohort who completed the second follow‐up and had baseline ECG data available. Exclusion criteria were missing or incomplete documentation of baseline characteristics or any key clinical variables required for ASCVD risk calculation. Participant selection is illustrated in Figure 1.

### 2.2 ECG Variables

The ECG parameters evaluated in this study included a range of continuous, binary, and categorical variables, categorized as heart rate and rhythm, P‐wave and atrial features, QRS and conduction features, ST‐T and repolarization features, and other relevant indices. Parameter definitions and diagnostic thresholds were established in accordance with standardized criteria derived from the Minnesota ECG Code Manual and its established definitions, as presented in Supporting Information S1: Table 1. Continuous parameters, including heart rate, P‐wave duration, QRS duration, and ST‐T intervals, were analyzed as recorded. Binary parameters were defined based on the presence or absence of specific ECG features, including arrhythmias, bundle branch blocks, and other findings, and were classified dichotomously.

### 2.3 ASCVD Risk Score

ASCVD risk was estimated using the pooled cohort equations, as incorporated within the American College of Cardiology (ACC)/American Heart Association (AHA) ASCVD Risk Estimator. The 10‐year risk score was calculated based on a composite of demographic and clinical variables, including age, sex, race, total cholesterol, high‐density lipoprotein (HDL) cholesterol, systolic blood pressure, diabetes status, antihypertensive treatment, and smoking history. ASCVD risk was modeled as a continuous variable, with higher scores reflecting a greater estimated 10‐year risk of developing atherosclerotic cardiovascular events.

### 2.4 Data Collection and Outcomes

Participant data were systematically extracted from the SHS electronic database by two physicians, including detailed information on demographic characteristics, medical history, and variables required for ASCVD risk score calculation. Three cardiology residents independently evaluated the electrocardiographic findings, and a senior interventional cardiologist adjudicated any discrepancies. Inter‐observer reliability was assessed using Cohen's kappa for categorical variables and intraclass correlation coefficients for continuous measures to ensure diagnostic consistency. A CVD event is defined as cardiovascular death, myocardial infarction (MI), stroke, or hospitalization due to cardiovascular events. All outcomes were adjudicated by a group of cardiologists outside the research team. All collected data were compiled and managed using Microsoft Excel for subsequent analysis.

### 2.5 Ethical Considerations

The study was approved by the Ethics Committee of Shiraz University of Medical Sciences (ethics code: IR.SUMS.MED.REC.1403.647) and conducted in accordance with the Declaration of Helsinki. All participant data were anonymized and handled with strict confidentiality.

### 2.6 Statistical Analysis

#### 2.6.1 Data Cleaning and Missingness

Continuous variables were summarized as means ± standard deviations, and categorical variables as counts and percentages. Missing data were addressed using complete‐case analysis; observations with missing values were excluded only for the affected variables, with < 5% missingness per group. All data preprocessing and analyses were performed in R (version 4.5.0) using the packages glmnet, pROC, and boot. A random seed was set (set.seed(1234)) to ensure reproducibility of resampling and cross‐validation procedures.

#### 2.6.2 Univariable Analysis and Variable Screening

All 63 ECG variables and 7 baseline characteristics were evaluated for association with cardiovascular events. Continuous variables were compared using independent samples *t*‐tests or Wilcoxon tests as appropriate; categorical variables were compared using chi‐square or Fisher's exact tests. Unadjusted *p* values from these analyses were corrected for multiple comparisons using the False Discovery Rate (Benjamini–Hochberg) method. Variables were considered for inclusion in multivariable models primarily based on clinical importance, prior literature, and relevance of key ECG and baseline characteristics. FDR‐adjusted *p* values (< 0.05) from univariable analyses were used as a supportive guide.

#### 2.6.3 Multivariable Model Building

Multivariable modeling was performed using two complementary strategies. First, a clinically pre‐specified logistic regression model was constructed, which included seven forced clinical confounders along with 15 ECG variables selected based on clinical relevance and univariable screening results. This model was fitted using both force entry of all 22 variables and a backward stepwise procedure guided by the Akaike Information Criterion (AIC). The number of predictors was chosen to satisfy the events‐per‐variable (EPV) rule, aiming for at least 10 events per predictor (221 events/22 predictors > 10). Second, to mitigate the risk of overfitting and to evaluate the robustness of variable selection, a penalized regression approach, the Least Absolute Shrinkage and Selection Operator (LASSO), was applied. Continuous variables were standardized, and 10‐fold cross‐validation was used to determine the optimal penalty parameter at both *λ*min and *λ*1se thresholds. Variables selected by LASSO were subsequently refitted in an unpenalized logistic regression model to obtain odds ratios (ORs) and 95% confidence intervals (CIs).

#### 2.6.4 Internal Validation and Sensitivity Analyses

Bootstrap resampling (1000 iterations) was performed to estimate 95% CIs for model AUCs and assess variable selection stability. Sensitivity analyses included refitting the model after excluding CVD history.

#### 2.6.5 Model Performance and Visualization

The discriminative ability of the final prespecified model was evaluated using ROC curves and AUC with 95% CI. Calibration was assessed with calibration plots and the Hosmer–Lemeshow test. Effect sizes and 95% CIs for predictors were displayed in forest plots. For the stepwise and LASSO models, the final predictor sets, along with their corresponding odds ratios and confidence intervals, are reported.

## 3 Results

### 3.1 Study Sample and Baseline Characteristics

A total of 442 participants were included (221 cases with a CVD event during 5‐year follow‐up and 221 event‐free controls). Matching on baseline ASCVD risk score was successful (mean ASCVD: 9.67 ± 7.57 in cases vs. 9.61 ± 7.46 in controls; *p*= 0.978). Baseline characteristics are summarized in Table 1. Despite similar ASCVD scores, several baseline variables differed between groups. Event cases were more likely to have diabetes (33% vs. 24%, *p* = 0.047), prior CVD (91.85% vs. 4.07%, *p* < 0.001), and a history of COPD (8% vs. 1%, *p* < 0.001). Controls were older on average than cases (63.93 ± 6.74 vs. 58.63 ± 7.05 years, *p* < 0.001) and had higher mean total cholesterol (194.7 ± 46.1 vs. 177.8 ± 47.3 mg/dL, *p* < 0.001) and a higher prevalence of current smoking (31% vs. 20%, *p* = 0.009). Other baseline variables (sex, SBP, DBP, HDL, LDL, alcohol use, HTN, history of cancer, chronic liver disease, chronic kidney disease) did not differ significantly between groups.

**Table 1:** Baseline clinical characteristics of event‐free controls and cardiovascular disease (CVD) cases in the Shiraz heart study cohort.

Variable	Control (event‐free) mean (SD)/*n* (%)	Case (event) mean (SD)/*n* (%)	*p* value
Age (year)	63.93 ± 6.74	58.63 ± 7.05	**< 0.001**
Sex (female)	72 (33%)	77 (35%)	0.7
SBP (mmHg)	126.07 (13.77)	126.64 (16.32)	0.618
DBP (mmHg)	80.07 (9.66)	79.64 (11.20)	0.696
Total cholesterol (mg/dL)	194.69 (46.1)	177.76 (47.31)	**< 0.001**
HDL‐C (mg/dL)	45.07 (11.38)	43.92 (9.95)	0.203
LDL‐C (mg/dL)	104.5 (28.90)	102.64 (31.23)	0.366
TG (mg/dL)	180.78 (116.34)	161.32 (86.6)	0.123
DM	55 (24%)	75 (33%)	**0.047**
HTN	89 (40%)	102 (46%)	0.249
Smoking	70 (31%)	45 (20%)	**0.009**
Alcohol consumption	15 (6%)	12 (5%)	0.682
ASCVD risk score	9.61 (7.46)	9.67 (7.57)	0.978
Previous CVD	9 (4.07%)	203 (91.85)	**< 0.001**
Hx of cancer	14 (6%)	5 (2%)	0.059
Hx of chronic liver disease	18 (8%)	9 (4%)	0.109
Hx of chronic kidney disease	9 (4%)	19 (8%)	0.081
Hx of COPD	2 (1%)	19 (8%)	**< 0.001**

*Note:* A *p*‐value below 0.05 is considered statistically significant.

Abbreviations: ASCVD, atherosclerotic cardiovascular disease; COPD, chronic obstructive pulmonary disease; DBP, diastolic blood pressure; DM, diabetes mellitus; HDL‐C, high‐density lipoprotein cholesterol; HTN, hypertension; Hx, history; LDL‐C, low‐density lipoprotein cholesterol; SBP, systolic blood pressure; TG, triglycerides.

### 3.2 Univariable ECG Screening

In univariable analyses of 63 ECG parameters, multiple conduction and repolarization markers showed robust associations with subsequent CVD events after FDR correction (Table 2). Cases had markedly shorter PR intervals (100 vs. 146 ms, FDR−*p *= 0.003) and longer QTc durations (451 vs. 423 ms, FDR−*p *= 0.003) compared to controls. Abnormal R‐wave progression (28% vs. 17%, FDR−*p *= 0.003), tall R waves in V1 (31% vs. 5%, FDR−*p *= 0.003), and evidence of left ventricular hypertrophy (50% vs. 11%, FDR−*p *= 0.003) were also significantly enriched in cases. Repolarization abnormalities were prominent, with a higher prevalence of inverted T‐waves (58% vs. 29%, FDR−*p *= 0.003), ST coving (13% vs. 4.4%, FDR−*p *= 0.003), and positive T‐waves in aVR (19% vs. 4.5%, FDR−*p *= 0.003) among cases. These variables were prioritized and subsequently incorporated into the multivariable modelling framework. The complete set of univariable results is provided in Supporting Information S1: Table 2.

**Table 2:** Baseline electrocardiographic (ECG) characteristics in event‐free controls and cardiovascular disease (CVD) cases.

Category	Variable	Control (event‐free) median (Q1, Q3)/*n* (%)	Case (event) median (Q1, Q3)/*n* (%)	*p* value (unadjusted)	*p* value (FDR‐adjusted)
Heart rate and rhythm	PR interval (ms)	146 (134, 162)	100 (90, 110)	< 0.001	**0.003**
P‐wave and atrial abnormalities	P‐wave duration (ms)	106 (96, 115)	110 (100, 116)	0.019	**0.046**
	Left atrial enlargement	37 (17%)	12 (5.4%)	< 0.001	**0.003**
QRS and conduction abnormalities	QRS duration (ms)	78 (71, 82)	96 (92, 100)	< 0.001	**0.003**
	QRS axis (°)	60 (48, 72)	24 (12, 36)	< 0.001	**0.003**
	RR interval changes (sinus arrhythmia)	33 (16%)	3 (1.4%)	< 0.001	**0.003**
	Fragmented QRS	36 (16%)	4 (1.8%)	< 0.001	**0.003**
	RSR' pattern	2 (0.9%)	12 (5.4%)	0.015	**0.038**
	rSr' pattern	12 (5.5%)	2 (0.9%)	0.014	**0.036**
	Abnormal R progression	38 (17%)	63 (28%)	< 0.001	**0.003**
	Tall R wave V1	10 (4.5%)	69 (31%)	< 0.001	**0.003**
	Tall R wave V2	39 (18%)	4 (1.8%)	< 0.001	**0.003**
	Low amplitude QRS	29 (13%)	59 (27%)	< 0.001	**0.003**
	LVH	24 (11%)	110 (50%)	< 0.001	**0.003**
	Notch on R	25 (11%)	9 (4.1%)	0.007	**0.02**
	Notch on S	35 (15.9%)	72 (32.5%)	< 0.001	**0.003**
ST‐T and repolarization abnormalities	QT interval (ms)	396 (380, 416)	430 (410, 448)	< 0.001	**0.003**
	QTc duration (ms)	422.84 (404.86, 438.27)	451.44 (421.25, 488)	< 0.001	**0.003**
	Tpeak‐Tend interval (ms)	70 (50, 80)	77 (54, 98)	< 0.001	**0.003**
	ST coving	9 (4.4%)	28 (13%)	< 0.001	**0.003**
	Inverted T wave	64 (29%)	128 (58%)	< 0.001	**0.003**
	Positive T wave in aVR	10 (4.5%)	42 (19%)	< 0.001	**0.003**
	U wave	35 (16%)	6 (2.7%)	< 0.001	**0.003**
Channelopathies and other findings	Terminal R in aVR	30 (13.5%)	68 (31%)	< 0.001	**0.003**
	Terminal S in V5 or V6	44 (20%)	14 (6.3%)	< 0.001	**0.003**

*Note:* A *p*‐value below 0.05 is considered statistically significant.

Abbreviations: aVR, augmented vector right lead; LVH, left ventricular hypertrophy; PR, PR interval; QRS, QRS complex; QT, QT interval; QTc, corrected QT interval; RR, RR interval; RSR', RSR prime pattern; rSr', rSr prime pattern; ST, ST segment; Tpeak–Tend, T‐peak to T‐end interval.

### 3.3 Multivariable Modeling of the Prespecified 22‐Variable Model

**Figure 2:**
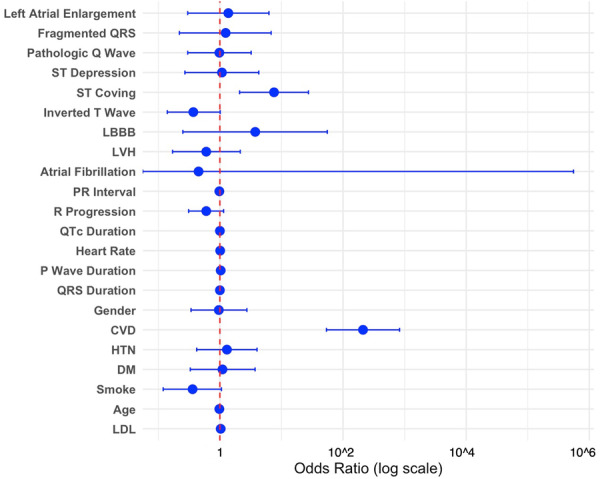
Multivariable associations of clinical and electrocardiographic features with CVD events in the pre‐specified model. Forest plot displaying odds ratios (blue circles) with 95% confidence intervals (horizontal lines) on a logarithmic scale for each predictor. The vertical dashed red line represents the null value (odds ratio = 1).

A prespecified logistic regression model was fitted, including seven clinical confounders and 15 ECG variables selected based on clinical relevance and univariable screening. The model satisfied the events‐per‐variable criterion (221 events/22 predictors > 10). Using a forced‐entry method, among the ECG predictors, ST‐segment coving (OR 7.57, 95% CI 2.09–27.4, *p*= 0.002) and PR interval (OR 0.98 per ms decrease, 95% CI 0.95–1.00, *p*= 0.036) remained statistically significant (Table 3). A forest plot was generated to visualize the odds ratios and 95% confidence intervals for all variables included in the main multivariable model, highlighting the relative strength and precision of each predictor (Figure 2). A backward stepwise selection procedure using the AIC yielded a more parsimonious model, which retained ST coving (OR 5.989, 95% CI 1.803–19.894, *p* = 0.0035) as a significant ECG predictor (Supporting Information S1: Table 3), confirming the robustness of the findings.

**Table 3:** Prespecified logistic regression model (forced‐entry) for predicting incident cardiovascular events.

Pre‐specified logistic regression model (forced entry)			
Variables	OR	95% CI	*p* value
Left atrial enlargement	1.37	0.30–6.26	0.682
Fragmented QRS	1.24	0.22–6.80	0.808
Pathologic Q wave	0.98	0.30–3.22	0.98
ST depression	1.08	0.27–4.28	0.911
ST coving	7.57	2.09–27.40	**0.002**
Inverted T wave	0.37	0.14–1.01	0.052
LBBB	3.75	0.25–55.52	0.337
LVH	0.6	0.17–2.14	0.428
Atrial fibrillation	0.45	0.00–554500	0.912
PR interval	0.98 (per ms)	0.95–1.00	**0.036**
R progression	0.6	0.31–1.15	0.125
QTc duration	1	0.99–1.01	0.418
Heart rate	1.01	0.97–1.06	0.537
P wave duration	1.03	1.00–1.07	0.087
QRS duration	1	0.98–1.02	0.945
Gender	0.96	0.34–2.74	0.938
CVD Hx	211.12	53.79–828.70	**< 0.001**
HTN	1.3	0.42–4.01	0.647
DM	1.1	0.33–3.73	0.875
Smoke	0.36	0.12–1.06	0.064
Age	0.98	0.92–1.05	0.528
LDL‐C	1.03	1.01–1.05	**0.002**

*Note:* A *p*‐value below 0.05 is considered statistically significant.

Abbreviations: CVD Hx, history of cardiovascular disease; DM, diabetes mellitus; HTN, hypertension; LBBB, left bundle branch block; LDL‐C, low‐density lipoprotein cholesterol; LVH, left ventricular hypertrophy; PR, PR interval; QTc, corrected QT interval.

### 3.4 Penalized Regression Using LASSO for Variable Selection

To reduce overfitting and identify a compact set of predictive features, we applied LASSO with 10‐fold cross‐validation. At the less conservative penalty (*λ*min), the LASSO model selected 13 predictors, including LDL, ST coving, abnormal R progression, tall R wave V2, Inverted T wave, notch on S, terminal S in V5/V6, QT interval, CVD Hx, PR interval, sinus tachycardia, and RAD. In the *λ*min model, several predictors yielded extreme odds ratios with wide confidence intervals following unpenalized logistic regression, indicating model instability and supporting the use of the more parsimonious *λ*1se model for primary inference. The *λ*1se model, which is more conservative, retained four variables including ST coving, PR interval, CVD Hx, and LDL. The four predictors were refit in an unpenalized logistic regression to obtain effect estimates, with ST coving (OR 4.31, 95% CI 1.38–13.0, *p*= 0.010) and PR interval (OR 0.977 per ms decrease, 95% CI 0.961–0.992, *p*= 0.003) emerging as significant predictors. The Supporting Information S1: Table 4 reports the full unpenalized refitted models of *λ*min and λ1se for exploratory purposes.

### 3.5 Internal Validation and Sensitivity Analyses

**Figure 3:**
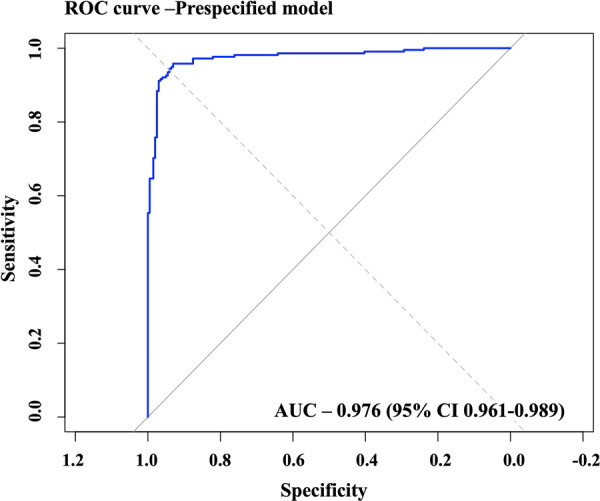
Discrimination of the pre‐specified model. Receiver operating characteristic (ROC) curve showing the discriminative performance of the pre‐specified model, with an area under the curve (AUC) of 0.976 (95% CI 0.961–0.989).

**Figure 4:**
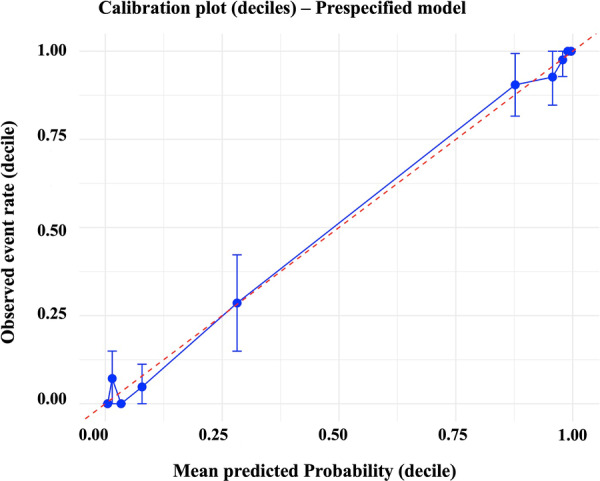
Calibration of the pre‐specified model. Calibration plot across deciles of predicted risk. The blue circles represent observed event rates with 95% confidence intervals, plotted against mean predicted probabilities. The solid blue line indicates the observed calibration, while the dashed red line represents the ideal 45° line (perfect calibration).

The main prespecified 22‐variable model demonstrated good discrimination, with an apparent AUC of 0.98 and a bootstrap‐corrected AUC of 0.97 (95% CI: 0.96–0.98) (Figure 3). As part of a sensitivity analysis, excluding CVD Hx due to extremely large and unstable effect estimates resulted in minimal change in model performance (21‐variable model, bootstrap AUC 0.94, 95% CI: 0.92–0.96). Similarly, the LASSO‐selected model with four predictors showed a bootstrap AUC of 0.96 (95% CI: 0.94–0.98), while the three‐predictor model without CVD Hx yielded an AUC of 0.88 (95% CI: 0.85–0.92), indicating that model discrimination remained robust across different variable selection approaches. Calibration of the main model was satisfactory. The Hosmer–Lemeshow test indicated no evidence of poor fit (*χ*² = 13.0, df = 8, *p*= 0.11), suggesting that predicted probabilities were consistent with observed outcomes. Visual inspection of the calibration plot confirmed good agreement between predicted and observed risk across deciles (Figure 4). The Brier score was 0.048, indicating high overall accuracy of the predicted probabilities (where 0 represents perfect prediction and 0.25 represents the worst performance for binary outcomes).

## 4 Discussion

In this nested case‐control study within the Shiraz Heart Study, we analyzed baseline ECGs from 221 participants who experienced CVD events over 5 years and 221 ASCVD risk–matched controls. Multivariable analyses, including prespecified logistic regression and LASSO modeling, identified ST‐segment coving and shorter PR interval as independent ECG predictors of incident cardiovascular events, providing prognostic information beyond traditional ASCVD risk factors.

Our findings extend prior observations that simple ECG markers can identify individuals at heightened cardiovascular risk. Previous studies, such as the LIFE and Strong Heart trials, demonstrated that ECG‐derived left ventricular hypertrophy and ST‐T abnormalities predicted heart failure, myocardial infarction, and cardiovascular mortality in higher‐risk cohorts [14, 15]. In contrast, our study focused on an ASCVD‐matched population with a broader risk spectrum, highlighting that ST‐segment coving and shorter PR interval are independently associated with incident cardiovascular events. Several other ECG features, including prolonged QTc, abnormal R‐wave progression, left ventricular hypertrophy, and T‐wave inversions, were significantly associated with events in univariable analyses, reflecting widespread conduction and repolarization abnormalities in cases.

ST‐segment coving is thought to reflect ischemia‐induced repolarization heterogeneity, where epicardial cells repolarize earlier than endocardial cells, creating voltage gradients that displace the ST segment upward on the ECG [16]. In our study, the prevalence of ST coving was significantly higher among participants who developed cardiovascular events, and it remained an independent predictor even after adjusting for ASCVD risk and clinical confounders. This finding is consistent with results from the Copenhagen ECG Study, which reported that ST‐segment deviations in the general population were associated with adverse prognosis, with ST elevation in lead V1 predicting increased cardiovascular mortality [17], and aligns with the ERICOECG cohort, where baseline ST‐segment abnormalities were associated with more than a two‐fold increased risk of myocardial infarction or death during follow‐up [18].

A shortened PR interval, which is an unusual ECG finding, most commonly reflects ventricular pre‐excitation via an accessory pathway (e.g., Wolff–Parkinson–White, which was assessed in our ECG evaluation) or accelerated atrioventricular (AV) conduction (e.g., Lown–Ganong–Levine or enhanced AV nodal conduction). Autonomic and endocrine states such as increased sympathetic tone or thyrotoxicosis can also shorten the PR interval by speeding AV nodal conduction [19, 20]. In our cohort, the PR interval was significantly shorter among individuals who developed cardiovascular events, and this association persisted after adjustment for ASCVD risk and clinical confounders. Longer PR intervals (first‐degree AV block) have been consistently linked to adverse outcomes, including higher incidence of atrial fibrillation, heart‐failure hospitalization, and mortality in large cohort studies and meta‐analyses [21, 22]. At the same time, several population studies report that risk is not strictly linear; very short PR intervals have also been associated with increased mortality or a U‐shaped relationship between PR duration and adverse outcomes, suggesting that both extremes of AV conduction time may carry prognostic significance [23, 24]. Taken together, these data suggest two plausible, non‐exclusive explanations for our finding: a subset of participants with short PR may have pre‐excitation or accessory‐pathway physiology that predisposes to malignant tachyarrhythmias, and short PR may also be a marker of heightened sympathetic or endocrine drive that augments arrhythmic and ischemic risk. In contrast, prolonged PR likely reflects conduction system disease and atrial remodeling that promote atrial fibrillation and heart failure events.

In our nested case–control cohort, the prespecified 22‐variable model demonstrated excellent discriminative performance, with a bootstrap‐corrected AUC of 0.97, while the more parsimonious LASSO‐selected four‐variable model, including ST coving and PR interval, maintained robust performance (AUC 0.96). These results indicate that even a small set of carefully selected ECG features can meaningfully enhance risk stratification. Comparable improvements in predictive performance have been reported in other populations [25]. For instance, artificial intelligence tools such as AIRE have increased the ECG‐based AUC for cardiovascular mortality to 0.832 [26], and deep learning models have improved the 5‐year concordance index to 0.79 [25]. Niu et al. [27] demonstrated that incorporating ECG markers into conventional risk scores increased the C‐statistic by approximately 0.06 [16]. Collectively, these findings highlight the value of integrating ECG‐derived parameters to refine cardiovascular risk prediction beyond traditional clinical scores.

Several ECG parameters reached statistical significance in univariable analyses, even when their clinical relevance seemed counterintuitive. This reflects the interdependence of ECG features, cohort heterogeneity, and confounding baseline characteristics. Variables such as P‐wave duration, left atrial enlargement, or low‐amplitude QRS may correlate with events in unadjusted analyses but lose predictive value once stronger ECG predictors and clinical covariates are included in the multivariable model. Multiple comparisons in high‐dimensional data can also produce spurious associations, which is why FDR correction and multivariable modeling were applied. Consequently, some statistically significant ECG variables may have odds ratios near 1, underscoring the difference between statistical and clinical significance. Sensitivity analysis excluding prior CVD history yielded unchanged model performance and conclusions, indicating that this variable did not affect the robustness of our results. Overall, our findings demonstrate that ECG‐derived parameters provide independent prognostic information for incident cardiovascular events. These results underscore the potential of combining traditional ECG analysis with advanced statistical or AI‐driven approaches to improve early risk stratification.

## 5 Strengths and Limitations

This study's strengths include its nested, risk‐matched case–control design within the Shiraz Heart Study, which enabled efficient analysis while accounting for baseline ASCVD risk scores. Rigorous ECG evaluation by multiple cardiologists enhanced diagnostic accuracy, and systematic assessment of a wide range of ECG parameters allowed identification of independent predictors through both prespecified and penalized modeling approaches. Additionally, the study provides novel cardiovascular risk data from a Middle Eastern population, helping to address an important regional evidence gap. At the same time, some limitations should be acknowledged. Its case–control design and relatively modest cohort size may limit statistical power, particularly for variables with low prevalence. Although cases and controls were matched on ASCVD risk, imbalances in some baseline characteristics remained, raising the possibility of residual confounding from both measured and unmeasured factors, including lifestyle and genetic variables. The reliance on baseline ECGs alone precludes assessment of temporal changes, and the observational design does not allow causal inference. Given the number of predictors relative to the sample size, some degree of model overfitting cannot be fully excluded. Although we mitigated this risk through prespecified modeling, LASSO penalization with cross‐validation, and extensive bootstrap internal validation, the possibility of residual overfitting remains. Moreover, generalizability is inherently limited because the cohort was drawn from a single‐center, ethnically homogeneous Iranian population. Consequently, our findings may not fully translate to more diverse clinical settings. External validation in larger, multi‐center, and ethnically varied cohorts is therefore essential to confirm the stability and broader applicability of the model. Additionally, model discrimination metrics such as AUC may appear higher in case‐control designs compared to prospective cohorts due to the enriched case proportion, potentially limiting direct comparability.

## 6 Conclusion

In this nested case–control study within the Shiraz Heart Study, we demonstrated that ST‐segment coving and shorter PR interval are independent predictors of incident cardiovascular events, providing incremental prognostic value beyond ASCVD risk scores. Both prespecified and penalized regression models confirmed these associations, and internal validation showed excellent discriminative performance. These findings underscore the potential of incorporating simple baseline ECG markers into risk prediction frameworks to enhance early identification of high‐risk individuals. External validation in larger and more diverse populations is warranted to confirm generalizability and support clinical implementation.

## Author Contributions

**Seyed Alireza Mirhosseini:** conceptualization, investigation, writing – original draft, methodology, writing – review and editing. **Pouria Azami:** conceptualization, investigation, writing – original draft, methodology, data curation, formal analysis. **Raziye Saeedizade:** investigation, data curation. **Mehrab Sayadi:** investigation, data curation, methodology, validation, formal analysis. **Mahya Beykihosseinabadi:** investigation, data curation. **Mohammad Keshavarz:** investigation, data curation. **Masood Dindari Parizi:** investigation, data curation. **Mahsa Borjzadehgashtaseb:** investigation, data curation. **Mohammadjavad Nobakhti:** investigation, data curation. **Armin Attar:** conceptualization, investigation, writing – original draft, supervision. **MohammadJavad Zibaeenezhad:** conceptualization, supervision, project administration, writing – review and editing.

## Funding

The authors received no specific funding for this work.

## Ethics Statement

This study was approved by the Ethics Committee of Shiraz University of Medical Sciences (reference number IR.SUMS.MED.REC.1403.647) and adhered to the ethical standards set forth by institutional and national research committees. It followed the principles of the 1964 Declaration of Helsinki and its subsequent amendments, or other equivalent ethical standards. Written informed consent was obtained from all participants involved in the study.

## Consent

Written informed consent was obtained from all participants involved in the study. The purpose of the research was fully explained to the patients, who were assured that their personal information would remain confidential and protected by the researcher.

## Conflicts of Interest

The authors declare no conflicts of interest.

## Transparency Statement

The corresponding author, Mohammad Javad Zibaeenez, affirms that this manuscript is an honest, accurate, and transparent account of the study being reported; that no important aspects of the study have been omitted; and that any discrepancies from the study as planned (and, if relevant, registered) have been explained.

## Supporting information


**Supplementary Table 1:** Detailed Definitions of ECG Variables. **Supplementary Table 2:** Comparison of Baseline Electrocardiographic (ECG) Parameters, Clinical Characteristics, and Comorbidities Between Event‐Free and Cardiovascular Event Cases.

## Data Availability

The data that support the findings of this study are available from the corresponding author upon reasonable request.
