# Supplementary material for: The Prognostic Ability of ECG Findings in Predicting Cardiovascular Events: A Five‐Year Nested Case—Cohort Study in an Iranian Population (Shiraz Heart Study)
Source: Health Sci Rep. 2026 Feb 28;9(3):e71830. doi: 10.1002/hsr2.71830 (PMC12949821; doi:10.1002/hsr2.71830)
Supplement: Supplementary file 1 — Supplementary Table 1: Detailed Definitions of ECG Variables. Supplementary Table 2: Comparison of Baseline Electrocardiographic (ECG) Parameters, Clinical Characteristics, and Comorbidities Between Event‐Free and Cardiovascular Event Cases. [file HSR2-9-e71830-s001.docx]

**Supplementary Document**

**The Prognostic Ability of ECG Findings in Predicting Cardiovascular Events: A Five-Year Nested Case–Cohort Study in an Iranian Population (Shiraz Heart Study)**

# **Authors**

Seyed Alireza Mirhosseini ^1,2 !^, Pouria Azami ^1,3,4 !^, Raziye Saeedizade ^3,4^, Mehrab Sayadi ^1^, Mahya Beykihosseinabadi ^5^, Mohammad Keshavarz ^3,4^, Masood Dindari Parizi ^3,4^, Mahsa Borjzadehgashtaseb ^3,4^, Mohammadjavad Nobakhti ^3,4^, Armin Attar ^3,4^, MohammadJavad Zibaeenezhad ^1,4*^

1. Cardiovascular Research Center, School of Medicine, Shiraz University of Medical Sciences, Shiraz, Iran

2. MD-MPH Department, School of Medicine, Shiraz University of Medical Sciences, Shiraz, Iran

3. School of Medicine, Shiraz University of Medical Sciences, Shiraz, Iran

4. Department of Cardiovascular Medicine, School of Medicine, Shiraz University of Medical Sciences, Shiraz, Iran

5. School of Medicine, Qazvin University of Medical Sciences, Qazvin, Iran

**! Seyed Alireza Mirhosseini and Pouria Azami contributed equally to this work and share first authorship.**

***Corresponding author: MohammadJavad Zibaeenezhad, MD**

**Email:** [zibaeem2@gmail.com](mailto:zibaeem2@gmail.com)

***Postal address:*** Department of Cardiovascular Medicine, School of Medicine, Zand Street, Shiraz University of Medical Sciences, Shiraz 71344-1864, Iran.

***Tel:*** +989173130918

***Fax:*** +987112349521

**Supplementary Table 1:** Detailed Definitions of ECG Variables

| Category | Variable name | Type (scale) | Definition |
| --- | --- | --- | --- |
| Heart Rate & Rhythm | Heart rate | Number (-) | Number of heart beats per minute |
|  | sinus bradychardia | Yes[1] or No[0] | 1: if HR < 60 |
|  | sinus tachycardia | Yes[1] or No[0] | 1: if HR > 100 |
|  | PR interval | Time (ms) | [LINK](https://litfl.com/pr-interval-ecg-library/) |
|  | PAC | Yes[1] or No[0] | [LINK](https://litfl.com/premature-atrial-complex-pac/) |
|  | PVC | Yes[1] or No[0] | [LINK](https://litfl.com/premature-ventricular-complex-pvc-ecg-library/) |
|  | atrial fibrillation | Yes[1] or No[0] | [LINK](https://litfl.com/atrial-fibrillation-ecg-library/) |
|  | atrial flutter | Yes[1] or No[0] | [LINK](https://litfl.com/atrial-flutter-ecg-library/) |
|  | non-sustained ventricular tachycardia | Yes[1] or No[0] | [LINK](https://litfl.com/ventricular-tachycardia-monomorphic-ecg-library/) |
| P-wave & Atrial Abnormalities | P-wave duration | Time (ms) | [LINK](https://litfl.com/p-wave-ecg-library/) |
|  | P-wave axis | Angle (degree) | [LINK](https://www.frontiersin.org/articles/10.3389/fcvm.2019.00053/full#:~:text=P%2DWave%20Axis,-P%2Dwave%20axis&text=It%20is%20determined%20by%20measuring,Figure%201)%20(31).) |
|  | prolonged p-wave | Yes[1] or No[0] | 1: if p-wave duration is > 120 |
|  | deep terminal negativity of p wave in V1 (biphasic p wave) | Yes[1] or No[0] | 1: Deep terminal negativity of P wave in V1 (DTNPV1) is defined as negative P prime larger than one small box (1 mm, or 0.1 mV) |
|  | left atrial enlargement | Yes[1] or No[0] | [LINK](https://litfl.com/left-atrial-enlargement-ecg-library/) |
|  | right atrial enlargement | Yes[1] or No[0] | [LINK](https://litfl.com/right-atrial-enlargement-ecg-library/) |
| QRS & Conduction Abnormalities | QRS duration | Time (ms) | [LINK](https://litfl.com/qrs-interval-ecg-library/) |
|  | RR interval changes (sinus arrhythmia) | Yes[1] or No[0] | 1: if RR interval changes were indicative of any sinus arrhythmias |
|  | QRS axis | Angle (degree) | [LINK](https://litfl.com/ecg-axis-interpretation/) |
|  | LAD | Yes[1] or No[0] | If the QRS axis is < -30 and > -90 |
|  | RAD | Yes[1] or No[0] | If the QRS axis is > +90 and < +180 |
|  | fragmented QRS | Yes[1] or No[0] | [LINK](https://www.researchgate.net/publication/232257016_Fragmented_QRS_What_is_the_meaning/figures?lo=1) |
|  | RSR' pattern | Yes[1] or No[0] | [LINK](https://www.researchgate.net/publication/232257016_Fragmented_QRS_What_is_the_meaning/figures?lo=1) |
|  | rSr' pattern | Yes[1] or No[0] | [LINK](https://www.researchgate.net/publication/232257016_Fragmented_QRS_What_is_the_meaning/figures?lo=1) |
|  | rSR’ pattern | Yes[1] or No[0] | [LINK](https://www.researchgate.net/publication/232257016_Fragmented_QRS_What_is_the_meaning/figures?lo=1) |
|  | R progression | Categorical [0] or [1] or [2] or [3] | 1: Early transition in V1 or V2  2: Normal transition in V3 or V4  3: Late transition in V5 or V6  0: Poor: no transition and R wave ≤ 3 mm in V3 |
|  | tall R wave V1 | Yes[1] or No[0] | 1: if a tall R wave in V1, defined as an R/S ratio of equal to or greater than one, was present |
|  | Tall R wave V2 | Yes[1] or No[0] | 1: if a tall R wave in V2, defined as an R/S ratio of equal to or greater than one, was present |
|  | high amplitude QRS | Yes[1] or No[0] | 1: Total 12-lead QRS amplitude more than 175 mm is a useful indicator of LV hypertrophy |
|  | low amplitude QRS | Yes[1] or No[0] | 1: if the amplitudes of all the QRS complexes in the limb leads are < 5 mm or the amplitudes of all the QRS complexes in the precordial leads are < 10 mm |
|  | RBBB | Yes[1] or No[0] | [LINK](https://litfl.com/right-bundle-branch-block-rbbb-ecg-library/) |
|  | LBBB | Yes[1] or No[0] | [LINK](https://litfl.com/left-bundle-branch-block-lbbb-ecg-library/) |
|  | incomplete RBBB | Yes[1] or No[0] | [LINK](https://litfl.com/right-bundle-branch-block-rbbb-ecg-library/) |
|  | incomplete LBBB | Yes[1] or No[0] | [LINK](https://litfl.com/left-bundle-branch-block-lbbb-ecg-library/) |
|  | SA block | Yes[1] or No[0] | [LINK](https://litfl.com/sinoatrial-exit-block-ecg-library/) |
|  | AV block | Yes[1] or No[0] | 1: in case of any type and degree of AV block |
|  | AV block type I | Yes[1] or No[0] | [LINK](https://litfl.com/first-degree-heart-block-ecg-library/) |
|  | AV block mobitz I | Yes[1] or No[0] | [LINK](https://litfl.com/av-block-2nd-degree-mobitz-i-wenckebach-phenomenon/) |
|  | AV block mobitz II | Yes[1] or No[0] | [LINK](https://litfl.com/av-block-2nd-degree-mobitz-ii-hay-block/) |
|  | CHB | Yes[1] or No[0] | [LINK](https://litfl.com/av-block-3rd-degree-complete-heart-block/) |
|  | LVH | Yes[1] or No[0] | [LINK](https://litfl.com/left-ventricular-hypertrophy-lvh-ecg-library/) |
|  | notch on R | Yes[1] or No[0] | [LINK](https://www.researchgate.net/publication/232257016_Fragmented_QRS_What_is_the_meaning/figures?lo=1) |
|  | notch on S | Yes[1] or No[0] | [LINK](https://www.researchgate.net/publication/232257016_Fragmented_QRS_What_is_the_meaning/figures?lo=1) |
| ST-T & Repolarization Abnormalities | QT interval | Time (ms) | [LINK](https://litfl.com/qt-interval-ecg-library/) |
|  | QTc duration | Time (ms) | Bazett formula: QTC = QT / √ RR  In case of bundle branch block: QTC – 0.5*QRS duration |
|  | prolonged QT interval | Yes[1] or No[0] | 1: for females if QTc > 460, for males if QTc > 450 |
|  | ST elevation | Yes[1] or No[0] | [LINK](https://litfl.com/st-segment-ecg-library/) |
|  | leads | string | please write the leads in which ST elevation was seen with Camma and a space.  Example: “I, II, aVF, aVL, V6” |
|  | territories | Categorical [0] or [1] or [2] | [0] No, [1] One territory: inferior (II, III, aVF), anterior (V1-V4), lateral (I, aVL, V5, V6), or aVR, [2] Multiple territories |
|  | ST depression | Yes[1] or No[0] | [LINK](https://litfl.com/st-segment-ecg-library/) |
|  | leads | string | please write the leads in which ST depression was seen with Camma and a space.  Example: “I, II, aVF, aVL, V6” |
|  | territories | Categorical [0] or [1] or [2] | [0] No, [1] One territory: inferior (II, III, aVF), anterior (V1-V4), lateral (I, aVL, V5, V6), or aVR, [2] Multiple territories |
|  | ST coving | Yes[1] or No[0] | [LINK](https://litfl.com/st-segment-ecg-library/) |
|  | leads | string | please write the leads in which ST coving was seen with Camma and a space.  Example: “I, II, aVF, aVL, V6” |
|  | territories | Categorical [0] or [1] or [2] | [0] No, [1] One territory: inferior (II, III, aVF), anterior (V1-V4), lateral (I, aVL, V5, V6), or aVR, [2] Multiple territories |
|  | inverted T wave | Yes[1] or No[0] | [LINK](https://litfl.com/t-wave-ecg-library/) |
|  | leads | string | please write the leads in which T inversion was seen with Camma and a space.  Example: “I, II, aVF, aVL, V6” |
|  | territories | Categorical [0] or [1] or [2] | [0] No, [1] One territory: inferior (II, III, aVF), anterior (V1-V4), lateral (I, aVL, V5, V6), or aVR, [2] Multiple territories |
|  | positive T wave in aVR | Yes[1] or No[0] | 1: presence of positive T wave in aVR |
|  | flat T wave | Yes[1] or No[0] | [LINK](https://litfl.com/t-wave-ecg-library/) |
|  | leads | string | please write the leads in which T wave flattening was seen with Camma and a space.  Example: “I, II, aVF, aVL, V6” |
|  | territories | Categorical [0] or [1] or [2] | [0] No, [1] One territory: inferior (II, III, aVF), anterior (V1-V4), lateral (I, aVL, V5, V6), or aVR, [2] Multiple territories |
|  | tent T wave | Yes[1] or No[0] |  |
|  | leads | string | please write the leads in which T wave tenting was seen with Camma and a space.  Example: “I, II, aVF, aVL, V6” |
|  | territories | Categorical [0] or [1] or [2] | [0] No, [1] One territory: inferior (II, III, aVF), anterior (V1-V4), lateral (I, aVL, V5, V6), or aVR, [2] Multiple territories |
|  | Tpeak-Tend interval | Time (ms) | Duration between peak of T wave to the end of T wave |
|  | T wave alternans | Yes[1] or No[0] | 1: T-wave alternans (TWA) refers to beat-to-beat fluctuations of T-wave amplitude and morphology |
|  | U wave | Yes[1] or No[0] | [LINK](https://litfl.com/u-wave-ecg-library/) |
| Pathologic Q waves & Ischemic Changes | Q/Qs wave abnormality | Yes[1] or No[0] | 1: any other type of q wave except for normal q waves:  Small Q waves are normal in most leads  Deeper Q waves (>2 mm) may be seen in leads III and aVR as a normal variant  Under normal circumstances, Q waves are not seen in the right-sided leads (V1-3) |
|  | leads | string | please write the leads in which q wave abnormality was seen with Camma and a space.  Example: “I, II, aVF, aVL, V6” |
|  | territories | Categorical [0] or [1] or [2] | [0] No, [1] One territory: inferior (II, III, aVF), anterior (V1-V4), lateral (I, aVL, V5, V6), or aVR, [2] Multiple territories |
|  | pathologic Q wave | Yes[1] or No[0] | 1: presence of pathologic Q wave:  > 40 ms (1 mm) wide  > 1 mm deep  > 25% of depth of QRS complex  Seen in leads V1-3 |
|  | leads | string | please write the leads in which pathologic q wave was seen with Camma and a space.  Example: “I, II, aVF, aVL, V6” |
|  | territories | Categorical [0] or [1] or [2] | [0] No, [1] One territory: inferior (II, III, aVF), anterior (V1-V4), lateral (I, aVL, V5, V6), or aVR, [2] Multiple territories |
| Channelopathies & Other Findings | Brugada | Yes[1] or No[0] | [LINK](https://litfl.com/brugada-syndrome-ecg-library/) |
|  | ARVD | Yes[1] or No[0] | [LINK](https://litfl.com/arrhythmogenic-right-ventricular-dysplasia-arvd/) |
|  | WPWs | Yes[1] or No[0] | [LINK](https://litfl.com/pre-excitation-syndromes-ecg-library/) |
|  | early repolarization | Yes[1] or No[0] | [LINK](https://litfl.com/benign-early-repolarisation-ecg-library/) |
|  | leads | string | If early repolarization is seen, please write the leads in which early repolarization was seen with Camma and a space.  Example: “I, II, aVF, aVL, V6” |
|  | territories | Categorical [0] or [1] or [2] | [0] No, [1] One territory: inferior (II, III, aVF), anterior (V1-V4), lateral (I, aVL, V5, V6), or aVR, [2] Multiple territories |
|  | terminal R in aVR | Yes[1] or No[0] | [LINK](https://litfl.com/r-wave-ecg-library/) |
|  | terminal S in V5 or V6 | Yes[1] or No[0] | [LINK](https://litfl.com/left-bundle-branch-block-lbbb-ecg-library/) |
|  | others | string | Please write any other significant finding or abnormality reported in site or seen in ECG |

ECG – electrocardiogram; bpm – beats per minute; PR – PR interval; PAC – premature atrial contraction; PVC – premature ventricular contraction; LAD – left axis deviation; RAD – right axis deviation; QRS – QRS complex; LBBB – left bundle branch block; RBBB – right bundle branch block; LVH – left ventricular hypertrophy; QT – QT interval; QTc – corrected QT interval; Tpeak-Tend – T-peak to T-end interval; CVD Hx – history of cardiovascular disease; HTN – hypertension; DM – diabetes mellitus; aVR – augmented vector right lead; WPWs – Wolff-Parkinson-White syndrome; ARVD – arrhythmogenic right ventricular dysplasia; FDR – false discovery rate.

**Supplementary Table 2:** Comparison of Baseline Electrocardiographic (ECG) Parameters, Clinical Characteristics, and Comorbidities Between Event-Free and Cardiovascular Event Cases

| **Category** | **Variable** | **Event-free Median (Q1, Q3) / n (%)** | **Event Median (Q1, Q3) / n (%)** | **p-value (unadjusted)** | **p-value (FDR-adjusted)** |  |
| --- | --- | --- | --- | --- | --- | --- |
|  |  |  |  |  |  |  |
| **Heart Rate & Rhythm** | **Heartrate (bpm)** | 68 (62, 76) | 67 (60, 75) | 0.3 | 0.404 |  |
|  | **PR interval (ms)** | 146 (134, 162) | 100 (90, 110) | <0.001 | **0.003** |  |
|  | **sinus bradychardia** | 38 (17%) | 53 (24%) | 0.1 | 0.171 |  |
|  | **sinus tachycardia** | 0 (0%) | 2 (0.9%) | 0.5 | 0.593 |  |
|  | **PAC** | 0 (0%) | 4 (1.8%) | 0.13 | 0.217 |  |
|  | **PVC** | 4 (1.8%) | 4 (1.8%) | >0.9 | 0.955 |  |
|  | **atrial fibrillation** | 3 (1.4%) | 3 (1.4%) | >0.9 | 0.955 |  |
|  | **atrial flutter** | 0 (0%) | 0 (0%) | 1 | 1 |  |
|  | **non-sustained ventricular tachycardia** | 0 (0%) | 0 (0%) | 1 | 1 |  |
| **P-wave & Atrial Abnormalities** | **P-wave duration (ms)** | 106 (96, 115) | 110 (100, 116) | 0.019 | **0.046** |  |
|  | **P-wave axis (°)** | 52 (37, 63.5) | 49 (32, 63) | 0.2 | 0.298 |  |
|  | **prolonged p-wave** | 44 (20%) | 34 (15%) | 0.3 | 0.404 |  |
|  | **deep terminal negativity of p wave in V1 (biphasic p wave)** | 31 (14%) | 23 (10%) | 0.3 | 0.404 |  |
|  | **left atrial enlargement** | 37 (17%) | 12 (5.4%) | <0.001 | **0.003** |  |
|  | **right atrial enlargement** | 8 (3.6%) | 15 (6.8%) | 0.2 | 0.298 |  |
| **QRS & Conduction Abnormalities** | **QRS duration (ms)** | 96 (88, 102) | 32 (15, 60) | <0.001 | **0.003** |  |
|  | **QRS axis (°)** | 96 (88, 102) | 33 (15, 60) | <0.001 | **0.003** |  |
|  | **RR interval changes (sinus arrhythmia)** | 33 (15.8%) | 3 (1.4%) | <0.001 | **0.003** |  |
|  | **LAD** | 16 (7.2%) | 11 (5.0%) | 0.4 | 0.5 |  |
|  | **RAD** | 7 (3.2%) | 0 (0%) | 0.022 | 0.051 |  |
|  | **fragmented QRS** | 36 (16%) | 4 (1.8%) | <0.001 | **0.003** |  |
|  | **RSR' pattern** | 2 (0.9%) | 12 (5.4%) | 0.015 | **0.038** |  |
|  | **rSr' pattern** | 12 (5.5%) | 2 (0.9%) | 0.014 | **0.036** |  |
|  | **rSR’ pattern** | 1 (0.5%) | 4 (1.8%) | 0.4 | 0.5 |  |
|  | **abnormal R progression** | 38 (17%) | 63 (28%) | <0.001 | **0.003** |  |
|  | **tall R wave V1** | 10 (4.5%) | 69 (31%) | <0.001 | **0.003** |  |
|  | **tall R wave V2** | 39 (18%) | 4 (1.8%) | <0.001 | **0.003** |  |
|  | **high amplitude QRS** | 15 (6.8%) | 29 (13%) | 0.039 | 0.08 |  |
|  | **low amplitude QRS** | 29 (13%) | 59 (27%) | <0.001 | **0.003** |  |
|  | **RBBB** | 4 (1.8%) | 4 (1.8%) | >0.9 | 0.955 |  |
|  | **LBBB** | 6 (2.7%) | 5 (2.3%) | >0.9 | 0.955 |  |
|  | **incomplete RBBB** | 11 (5.0%) | 22 (10.0%) | 0.07 | 0.129 |  |
|  | **incomplete LBBB** | 16 (7.2%) | 5 (2.3%) | 0.025 | 0.056 |  |
|  | **SA block** | 3 (1.4%) | 3 (1.4%) | >0.9 | 0.955 |  |
|  | **AV block** | 11 (5.0%) | 3 (1.4%) | 0.057 | 0.108 |  |
|  | **AV block type I** | 8 (3.6%) | 0 (0%) | 0.013 | **0.035** |  |
|  | **AV block mobitz I** | 0 (0%) | 0 (0%) | 1 | 1 |  |
|  | **AV block mobitz II** | 0 (0%) | 0 (0%) | 1 | 1 |  |
|  | **CHB** | 0 (0%) | 5 (2.3%) | 0.072 | 0.129 |  |
|  | **LVH** | 24 (11%) | 110 (50%) | <0.001 | **0.003** |  |
|  | **notch on R** | 25 (11%) | 9 (4.1%) | 0.007 | **0.02** |  |
|  | **notch on S** | 35 (15.9%) | 72 (32.5%) | <0.001 | **0.003** |  |
| **ST-T & Repolarization Abnormalities** | **QT interval (ms)** | 396 (380, 416) | 430 (410, 448) | <0.001 | **0.003** |  |
|  | **QTc duration (ms)** | 422.84 (404.86, 438.27) | 451.44 (421.25, 488) | <0.001 | **0.003** |  |
|  | **Tpeak-Tend interval (ms)** | 70 (50, 80) | 77 (54, 98) | <0.001 | **0.003** |  |
|  | **prolonged QT interval** | 20 (9.0%) | 8 (3.6%) | 0.032 | 0.07 |  |
|  | **ST elevation** | 28 (13%) | 15 (6.8%) | 0.054 | 0.105 |  |
|  | **ST depression** | 45 (20%) | 28 (13%) | 0.04 | 0.08 |  |
|  | **ST coving** | 9 (4.4%) | 28 (13%) | <0.001 | **0.003** |  |
|  | **inverted T wave** | 64 (29%) | 128 (58%) | <0.001 | **0.003** |  |
|  | **positive T wave in aVR** | 10 (4.5%) | 42 (19%) | <0.001 | **0.003** |  |
|  | **flat T wave** | 65 (29%) | 49 (22%) | 0.1 | 0.171 |  |
|  | **tent T wave** | 4 (1.8%) | 1 (0.5%) | 0.4 | 0.5 |  |
|  | **T wave alternans** | 1 (0.5%) | 4 (1.8%) | 0.4 | 0.5 |  |
|  | **U wave** | 35 (16%) | 6 (2.7%) | <0.001 | **0.003** |  |
| **Pathologic Q waves & Ischemic Changes** | **Q/Qs wave abnormality** | 73 (33%) | 62 (28%) | 0.3 | 0.404 |  |
|  | **pathologic Q wave** | 59 (27%) | 40 (18%) | 0.04 | 0.08 |  |
| **Channelopathies & Other Findings** | **Brugada** | 8 (3.6%) | 12 (5.4%) | 0.5 | 0.593 |  |
|  | **ARVD** | 0 (0%) | 1 (0.5%) | >0.9 | 0.955 |  |
|  | **WPWs** | 1 (0.5%) | 5 (2.3%) | 0.2 | 0.298 |  |
|  | **early repolarization** | 19 (8.6%) | 11 (5.0%) | 0.2 | 0.298 |  |
|  | **terminal R in aVR** | 30 (13.5%) | 68 (31%) | <0.001 | **0.003** |  |
|  | **terminal S in V5 or V6** | 44 (20%) | 14 (6.3%) | <0.001 | **0.003** |  |
| **Comorbidities** | **CVD Hx** | 9 (4.3%) | 203 (92%) | <0.001 | **0.003** |  |
| **Demographics** | **Age (yr)** | 57 (51, 63) | 59 (53, 65) | 0.003 | **0.009** |  |
|  | **Sex (Female)** | 72 (33%) | 77 (35%) | 0.7 | 0.817 |  |
| **Clinical Risk Factors** | **LDL-C (mg/dL)** | 104 (85, 122) | 98 (79, 121) | 0.5 | 0.593 |  |
|  | **HTN** | 87 (39%) | 100 (45%) | 0.3 | 0.404 |  |
|  | **DM** | 61 (28%) | 94 (43%) | 0.002 | **0.006** |  |
|  | **Smoking** | 70 (32%) | 44 (20%) | 0.007 | **0.02** |  |

ECG – electrocardiogram; bpm – beats per minute; PR – PR interval; PAC – premature atrial contraction; PVC – premature ventricular contraction; LAD – left axis deviation; RAD – right axis deviation; QRS – QRS complex; LBBB – left bundle branch block; RBBB – right bundle branch block; LVH – left ventricular hypertrophy; QT – QT interval; QTc – corrected QT interval; Tpeak-Tend – T-peak to T-end interval; CVD Hx – history of cardiovascular disease; HTN – hypertension; DM – diabetes mellitus; aVR – augmented vector right lead; WPWs – Wolff-Parkinson-White syndrome; ARVD – arrhythmogenic right ventricular dysplasia; FDR – false discovery rate.

**Supplementary Table 3:** Pre-specified Logistic Regression Model (Backward Stepwise Selection Based on Akaike Information Criterion, AIC) for Predictors of Cardiovascular Events

| **Pre-specified logistic regression model (backward stepwise, AIC)** | | | |
| --- | --- | --- | --- |
| **Term** | **OR** | **95% CI** | **p-value** |
| **ST coving** | 5.989 | 1.803–19.894 | **0.0035** |
| **inverted T wave** | 0.4006 | 0.1575–1.019 | 0.0548 |
| **PR interval** | 0.9831 | 0.9654–1.0011 | 0.0654 |
| **abnormal R progression** | 0.5815 | 0.3184–1.0618 | 0.0776 |
| **P wave duration** | 1.028 | 0.9977–1.0583 | 0.0709 |
| **CVD Hx** | 141.5 | 42.74–468.36 | **5.12E-16** |
| **Smoke** | 0.3529 | 0.1283–0.9705 | 0.0436 |
| **LDL-C** | 1.026 | 1.009–1.0429 | **0.002** |

Stepwise logistic regression was performed as a sensitivity analysis using Akaike Information Criterion (AIC) in both directions, starting from the prespecified full model. The final model included 8 predictors: STcoving, invertedTwave, PRinterval, Rprogression, Pwaveduration, CVDHx, Smoke, and LDL. The analysis used 415 complete-case observations. Model fit statistics were: AIC = 173.3, null deviance = 576.23 (df = 415), residual deviance = 155.30 (df = 407). Binary variables were coded as 0/1. Compared with the prespecified model, several variables were removed during stepwise selection.

ST – ST segment; PR – PR interval; CVD Hx – history of cardiovascular disease; LDL-C – low-density lipoprotein cholesterol; OR – odds ratio; CI – confidence interval.

**Supplementary Table 4:** LASSO-Selected Logistic Regression Models for Cardiovascular Event Prediction (A. λmin – less conservative; B. λ1SE – more conservative)

| **A. λmin LASSO model (less conservative)** | | | |
| --- | --- | --- | --- |
| **Variable** | **OR (Estimate)** | **95% CI** | **p-value** |
| **CVD Hx** | 142 | 46.1 – 527 | **<0.001** |
| **LDL-C** | 1.02 | 1.01 – 1.04 | 0.005 |
| **ST coving** | 3.81 | 1.03 – 13.9 | 0.043 |
| **R progression** | 0.54 | 0.28 – 1.05 | 0.065 |
| **Tall R wave V2** | 0.24 | 0.04 – 1.29 | **0.118** |
| **Inverted T wave** | 0.54 | 0.21 – 1.41 | 0.205 |
| **Notch on S** | 1.12 | 0.54 – 2.36 | 0.76 |
| **Terminal S in V5/V6** | 0.97 | 0.30 – 3.09 | 0.953 |
| **QT interval** | 1 | 0.99 – 1.02 | **0.381** |
| **PR interval** | 0.99 | 0.98 – 1.02 | 0.642 |
| **Sinus tachycardia** | 9.8 × 10⁷ | Unstable (NA) | **0.993** |
| **RAD** | 7.6 × 10⁻⁸ | Unstable (NA) | 0.99 |
|  |  |  |  |
| **B. λ1SE LASSO model (more conservative)** | | | |
| **Variable** | **OR** | **95% CI** | **p-value** |
| **CVDHx** | 205 | 77.1 – 637 | <0.001 |
| **PR interval** | 0.977 | 0.961 – 0.992 | 0.003 |
| **ST coving** | 4.31 | 1.38 – 13.0 | 0.01 |
| **LDL-C** | 1.02 | 1.01 – 1.04 | **0.008** |

Penalized logistic regression using the Least Absolute Shrinkage and Selection Operator (LASSO) was performed to identify a compact set of predictive ECG and clinical variables while minimizing overfitting. Two models were generated based on cross-validated penalty parameters: **λmin (less conservative)** and **λ1SE (more conservative)**.

**A. λmin LASSO model (less conservative):** This model used the penalty parameter that minimized cross-validated error, allowing inclusion of a larger number of predictors. Eleven variables were retained, including CVD history, LDL-C, ST coving, R progression, tall R wave V2, inverted T wave, notch on S, terminal S in V5/V6, QT interval, PR interval, and sinus tachycardia/RAD. Some estimates were unstable (e.g., sinus tachycardia, RAD), reflecting limited events for rare predictors. Effect estimates (ORs), 95% confidence intervals, and p-values are reported for complete-case observations (n = 415).

**B. λ1SE LASSO model (more conservative):** This model used a penalty parameter within one standard error of the minimum cross-validated error to produce a sparser, more stable model. Four predictors were retained: CVD history, PR interval, ST coving, and LDL-C. Effect estimates (ORs), 95% confidence intervals, and p-values are reported for complete-case observations (n = 415). This conservative model provides a robust set of variables for primary inference while mitigating overfitting.

CVD Hx – history of cardiovascular disease; LDL-C – low-density lipoprotein cholesterol; ST – ST segment; PR – PR interval; RAD – right axis deviation; OR – odds ratio; CI – confidence interval; λmin – penalty parameter yielding minimum cross-validated error; λ1SE – penalty parameter yielding the most regularized model within one standard error of minimum.
